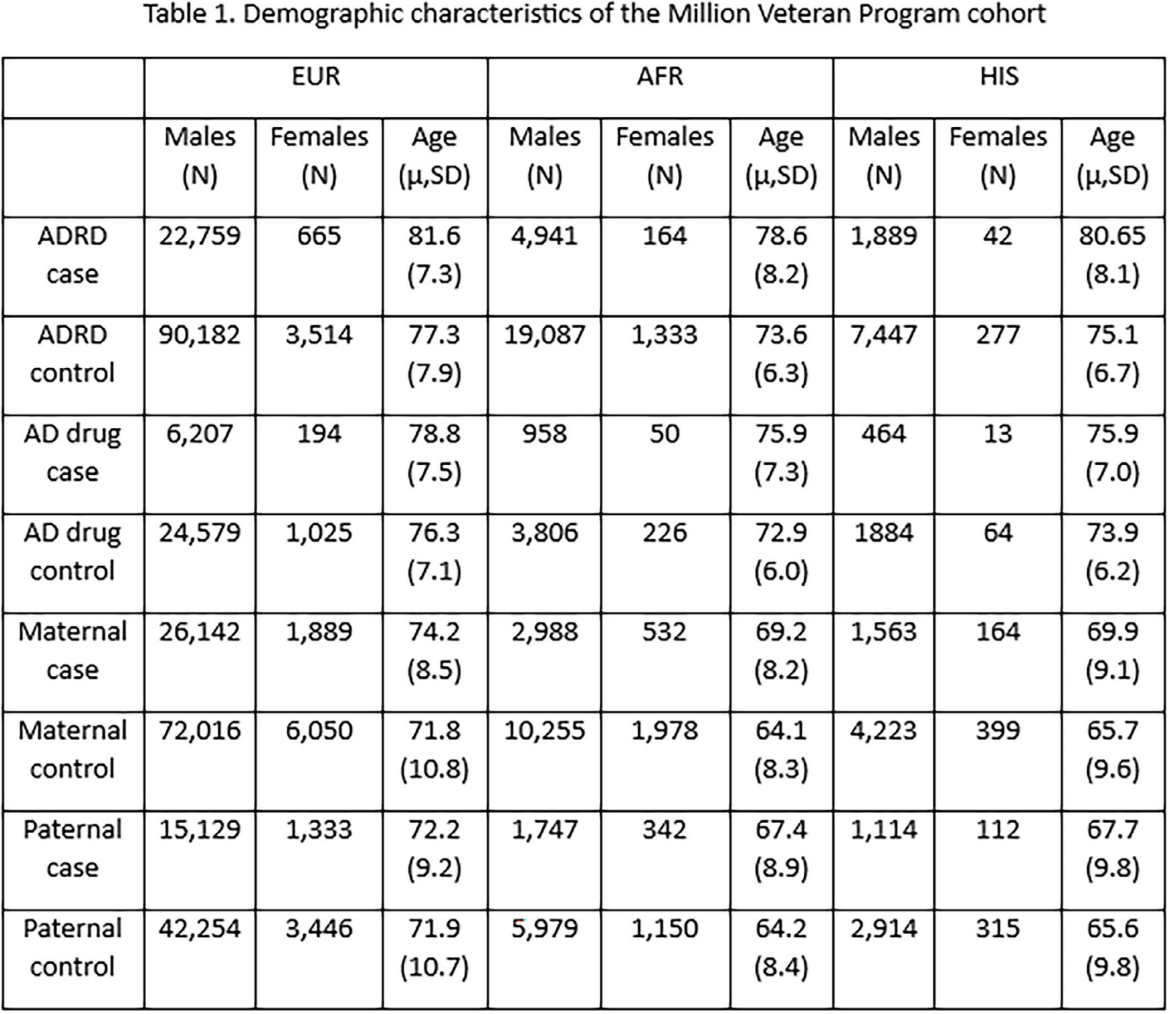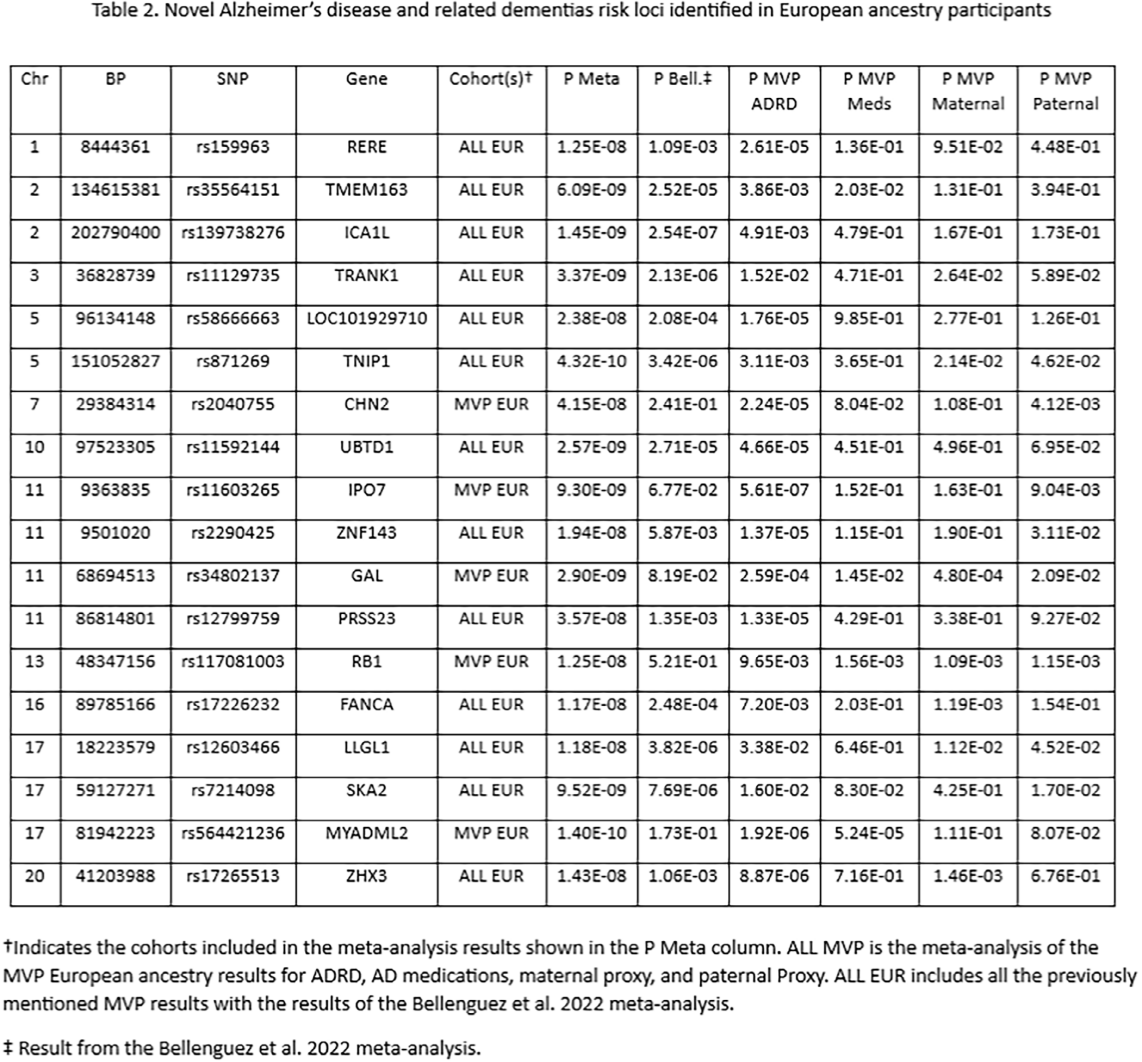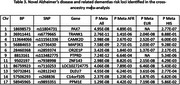# GWAS of 205,500 Alzheimer's disease and related dementia cases reveals 19 novel, European specific and 11 cross‐ancestry risk loci

**DOI:** 10.1002/alz70855_105500

**Published:** 2025-12-24

**Authors:** Richard Sherva, Henry Bayly, Rui Zhang, Jesse Mez, Richard L. Hauger, Victoria C. Merritt, Matthew S. Panizzon, J. Michael Gaziano, Lindsay A. Farrer, Mark W. Logue, VA Million Veteran Program

**Affiliations:** ^1^ Biomedical Genetics, Department of Medicine, Boston University Medical School, Boston, MA, USA; ^2^ National Center for PTSD, VA Boston Healthcare System, Boston, MA, USA; ^3^ Boston University, Boston, MA, USA; ^4^ Department of Neurology, Boston University Chobanian & Avedisian School of Medicin, Boston, MA, USA; ^5^ Boston University Chronic Traumatic Encephalopathy Center, Boston, MA, USA; ^6^ Boston University Chobanian & Avedisian School of Medicine, Boston, MA, USA; ^7^ Center for Behavior Genetics of Aging, University of California, San Diego, La Jolla, CA, USA; ^8^ Department of Psychiatry, University of California San Diego, La Jolla, CA, USA; ^9^ Center of Excellence for Stress and Mental Health, VA San Diego Healthcare System, San Diego, CA, USA; ^10^ Center for Excellence for Stress and Mental Health (CESAMH), VA San Diego Healthcare System, San Diego, CA, USA; ^11^ Veterans Affairs San Diego Healthcare System, La Jolla, CA, USA; ^12^ University of California San Diego, La Jolla, CA, USA; ^13^ Division of Aging, Brigham & Women's Hospital, Harvard Medical School, Boston, MA, USA; ^14^ Million Veteran Program (MVP) Coordinating Center, VA Boston Healthcare System, Boston, MA, USA; ^15^ Department of Neurology and Ophthalmology, Boston University Chobanian & Avedisian School of Medicine, Boston, MA, USA; ^16^ Department of Neurology, Boston University Chobanian & Avedisian School of Medicine, Boston, MA, USA; ^17^ Framingham Heart Study, Boston University Chobanian & Avedisian School of Medicine, Boston, MA, USA; ^18^ Bioinformatics Program, Boston University, Boston, MA, USA; ^19^ Department of Biostatistics, Boston University School of Public Health, Boston, MA, USA; ^20^ Department of Psychiatry, Boston University Chobanian & Avedisian School of Medicine, Boston, MA, USA

## Abstract

**Background:**

As biobank‐scale cohorts emerge, there has been a great increase in the number of dementia cases available for genetic meta‐analyses, resulting in a substantial increase in the number of dementia risk variants identified and replicated. Although also increasing, the number of non‐European ancestry participants in most of these studies is still substantially lower. Although primarily European ancestry (EUR), the Million Veteran Program (MVP) contains a substantial proportion of African (AFR) and Hispanic (HIS) ancestry participants. Here, we analyzed three dementia phenotypes in MVP, and meta‐analyzed them with publicly available dementia summary statistics.

**Methods:**

Three phenotypes were analyzed in MVP (Table 1): 1) Alzheimer's disease and related dementias (ADRD), defined by two or more ADRD treatment codes in electronic medical records (EMR); 2) prescriptions for medications to treat dementia (e.g. cholinesterase inhibitors), also from EMR; 3) maternal and paternal proxy dementia as reported in MVP surveys. These analyses were independent, with non‐overlapping case/control sets. We first performed GWAS of these phenotypes within MVP ancestry groups (EUR, AFR, HIS), adjusting for sex and ancestry principal components. These were used along with Bellenguez et al. 2022, and AFR statistics from Kunkle et al. 2021 to generate a EUR MVP meta‐analysis, an MVP/Bellenguez EUR meta‐analysis, and a cross‐ancestry meta‐analysis using MR Mega. Novel regions were defined as genome‐wide significant (GWS) loci at least 250KB from the peak SNP in previous GWASs, and if within 1MB of that SNP, had a pairwise r^2^<0.2.

**Results:**

We identified 19 novel, GWS regions in EUR, five in MVP EUR, and 14 in the meta‐analysis (Table 2). We identified 11 novel regions in the cross‐ancestry meta. Two genes, *PAX7* and *PTPRD* (not novel, but previously only observed in HIS), were unique to the AFR and HIS cohorts (Table 3).

**Conclusion:**

The MVP‐specific genes may be involved in processes related to more general (not AD specific) dementia pathways or have stronger effects in males. Several of the novel genes identified in EUR and cross‐ancestry meta‐analysis are involved in neuronal development and function or have been linked to cognitive outcomes.